# Hypermethylation of the *PZP* gene is associated with hepatocellular carcinoma cell proliferation, invasion and migration

**DOI:** 10.1002/2211-5463.13093

**Published:** 2021-02-21

**Authors:** Minhua Wu, Hui Lan, Zhongwei Ye, Yonghui Wang

**Affiliations:** ^1^ Department of Medical Oncology Lishui Municipal Central Hospital Zhejiang Province China

**Keywords:** HCC, invasion and migration, methylation, proliferation, PZP

## Abstract

Pregnancy zone protein (PZP), a member of the proteinase inhibitor I39 (‐2‐macroglobulin) family of proteins, is involved in the initiation and development of various tumors. The gene encoding PZP is hypermethylated and expressed at low levels in hepatocellular carcinoma (HCC) tissue and cells, but the function of PZP in HCC cells remains unclear. Here, we analyzed DNA methylation and mRNA expression of HCC in The Cancer Genome Atlas Liver Hepatocellular Carcinoma dataset. We identified 10 methylation‐driven genes, of which *PZP* was significantly hypermethylated and poorly expressed in tumor tissue. We confirmed that *PZP* is highly methylated and poorly expressed in HCC cell lines via quantitative real‐time PCR experiment and methylation‐specific PCR. Furthermore, PZP markedly inhibited the proliferation, invasion and migration of HCC cells. These findings may provide a basis for exploring novel therapeutic targets for HCC.

## Graphical abstract

We analyzed DNA methylation and mRNA expression of hepatocellular carcinoma (HCC) in the TCGA‐LIHC dataset. We identified 10 methylation‐driven genes, of which pregnancy zone protein (PZP) was significantly hypermethylated and poorly expressed in tumor tissue. We confirmed that PZP is highly methylated and poorly expressed in HCC cell lines via RT‐PCR and methylation‐specific PCR. Furthermore, PZP markedly inhibited the proliferation, invasion and migration of HCC cells.

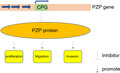

**Abbreviations**
5‐aza‐dC: 5‐aza‐2ʹ‐deoxycytidine
CGI: CpG island
DEmRNA: differentially expressed mRNA
FC: fold change
HCC: hepatocellular carcinoma
MSP: methylation‐specific PCR
MTT: 3‐(4,5‐dimethylthiazol‐2‐yl)‐2,5‐diphenyl‐tetrazolium bromide
NC: negative control
PZP: pregnancy zone protein
SD: standard deviation
TCGA‐LIHC: The Cancer Genome Atlas Liver Hepatocellular Carcinoma

Liver cancer is the sixth most common cancer and the fourth most common cause of cancer‐related deaths in the world [1]. Hepatocellular carcinoma (HCC) is the main histological subtype of liver cancer, accounting for 90% of primary liver cancer, yet its pathogenesis remains unclear. With the deeper research on tumors, it has been reported that the two mechanisms associated with the occurrence and development of tumors are genetic variation and epigenetic modification [2]. Currently, the epigenetic mechanism that has been most frequently researched mainly focuses on DNA methylation and histone modification. DNA methylation has been the first epigenetic phenomenon [3]. People usually refer to the covalent addition of a methyl (CH3) group to the C5 position of the cytosine pyrimidine ring (usually in CpG dinucleotides) as DNA methylation [4, 5]. The CpG site exists in two forms: one being dispersed in DNA sequence, and another being highly concentrated in large clusters called CpG islands (CGIs) (CpG > 50%, length > 200 base pairs) [6]. At present, DNA methylation, especially the aberrant CGI methylation in the gene promoter region, has become the hotspot of tumor research [7]. Aberrant CGI methylation of the gene promoter region can activate proto‐oncogenes or silence tumor suppressor genes, leading to altered expression of downstream key genes and consequently promoting the aberrant proliferation of cancer cells [6]. A number of research studies have unveiled that aberrant DNA methylation is able to facilitate tumor cell proliferation, invasion and migration of colorectal cancer [8], gastric cancer [9], liver cancer [10, 11], HCC [12] and so on.

This study found that pregnancy zone protein (PZP) in HCC tissue and cells was hypermethylated and poorly expressed. PZP is a protein coding gene that is capable of suppressing all four classes of proteinases by a unique ‘trapping’ mechanism. Devriendt *et al*. [13] cloned the full‐length cDNA of *PZP* in HCC in 1991. Subsequently, research has indicated that PZP is associated with the liver fibrosis in nonalcoholic fatty liver [14], while liver fibrosis is the beginning of normal liver tissue gradually transferring to cirrhotic tissue. Nevertheless, the functional mechanism of PZP in HCC cells remains unclear, and there is an urgent need to carry out further investigation.

This study initially analyzed data of methylation and mRNA expression of HCC through The Cancer Genome Atlas Liver Hepatocellular Carcinoma (TCGA‐LIHC) database, finding that the down‐regulation of PZP in HCC was caused by promoter hypermethylation. After that, we explored the relationship between *PZP* methylation level and *PZP* expression in HCC cell proliferation, migration, invasion and apoptosis through *in vitro* cell experiments and further clarified the mechanism of *PZP* regulating the development of HCC.

## Materials and methods

### Data source and processing

HCC data, including methylation data, mRNA expression profiles and clinical information, were downloaded from the TCGA‐LIHC dataset. A total of 424 mRNA expression profiles (normal: *n* = 50, tumor: *n* = 374) and methylation data of 430 samples (normal: *n* = 50, tumor: *n* = 380) were obtained. Methylation data were standardized using the ‘limma’ package. Differential analysis was conducted to screen the differentially expressed mRNAs (DEmRNAs) using the ‘edgeR’ package (|logFC| > 2, *P*_adj_ < 0.05). Candidate methylation‐driven genes were screened using the ‘MethylMix’ package (|logFC| > 0.5, *P*_adj_ < 0.05, Correlation coefficient < −0.3). The ‘survival’ package was used to analyze the effect of the methylation and expression levels of the target methylation‐driven gene on a patient's prognosis.

### Cell culture

This study used the human liver cell line HL‐7702 (3131C0001000200006) provided by Shanghai Institutes for Biological Sciences, Chinese Academy of Sciences, and the human HCC cell lines Hep G2 (3111C0001CCC000035), Hep3b (3111C0001CCC000376), HuH‐7 (3111C0001CCC000679), Li‐7 (3111C0001CCC000678) and Hep 3B2.1‐7 (3111C0001CCC000474) provided by Cell Source Center, Institute of Basic Medical Sciences, Chinese Academy of Medical Sciences. The cells were cultured at 37 °C in a humid environment containing 5% CO_2_ according to the manufacturer’s instructions.

### DNA extraction and sodium bisulfite modification

QIAamp DNA Mini Kit (50) (51304; Qiagen, Hilden, Germany) was used to extract the genomic DNA of the human normal liver cell line and the five human HCC cell lines according to the instructions. Thereafter, the genomic DNA was processed by sodium bisulfite: 2 μg DNA was dissolved in 50 μL deionized water, denatured in 0.2 mol·L^−1^ NaOH at 50 °C for 10 min and water bathed in fresh 30 μL 10 mmol·L^−1^ hydroquinone and 520 μL sodium bisulfite (3.6 mol·L^−1^, pH 5.0) at 50 °C for 18 h. After being purified in adsorption column, it was precipitated in absolute alcohol and dissolved in 50 μL deionized water.

### Methylation‐specific PCR

Methylation‐specific PCR (MSP) was used for amplification of the *PZP* gene sequence. Primers were designed according to the *PZP* gene sequence (Gene ID: 5858) and synthesized by MethPrimer. The 25 μL PCR system was as follows: 10× buffer 2.5 μL, dNTP 2.0 μL, TaqE 0.5 μL, double‐distilled H_2_O 17.5 μL, Pmix 1 μL, MgSO_4_ 0.5 μL and DNA 1 μL. Thirty‐five cycles began after predenaturation at 94 °C for 3 min: 94 °C 30 s, 71 °C 1 min, 75 °C 45 s. At last, extension was processed at 72 °C for 7 min (preserved at 4 °C). PCR products were loaded on 2% agarose gel electrophoresis and then observed. Primer sequences are listed in Table S1.

### Real‐time quantitative PCR

According to the manufacturer's instructions, TRIzol reagent (Invitrogen, Carlsbad, CA, USA) was used to extract total RNA from HCC tissue and cells. Then RNA was transcribed into cDNA by using the Reverse Transcription Assay Kit (Invitrogen). Quantitative real‐time PCR was performed on an ABI 7900HT instrument (Applied Biosystems, Foster City, CA, USA) using the miScript SYBR Green PCR Kit (Qiagen). GAPDH was used as internal reference, and each measurement was normalized to GAPDH expression. The quantitative value was expressed using the $2^{‐\Delta \Delta C_{t}}$ method to compare the differences of target gene expression in the control group and the test group. We repeated the experiment in triplicate. Primer sequences are listed in Table S1.

### Demethylation experiment

For the demethylation experiment, all the cell lines were processed using 2.5 μmol·L^−1^ demethylating agent 5‐aza‐2′‐deoxycytidine (5‐aza‐dC) for 6 days. Subsequently, cells were collected, and total RNA was extracted to detect PZP expression.

### Construction of pCDH‐*PZP* recombinant plasmid

The cDNAs of HuH‐7 and Hep G2 cells were used as templates to amplify the human full‐length PZP coding sequence. pCDH‐CMV‐MCS‐EF1‐Puro (YB‐0879; Shanghai Yu Bo Biotech Co., Ltd., Shanghai, China) lentivirus vector was used to construct PZP overexpression plasmid. Packed lentiviral supernatant was added to the growth medium of HL‐7702 and Hep G2 cells, and Puromycin was used to screen stably transfected cell lines.

### MTT assay

Cells in the negative control (NC) group and *PZP* overexpression group were digested with 0.25% trypsin and seeded in a 96‐well plate at a density of 2 × 10^3^ cells per well. Three repeated wells were set for each treatment. Each well was added with 200 μL RPMI 1640 medium, which contained 10% FBS. At 0, 1, 2, 3, 4 and 5 days, 20 μL 3‐(4,5‐dimethylthiazol‐2‐yl)‐2,5‐diphenyl‐tetrazolium bromide (MTT; 5 g·L^−1^) was added into the cells, and then the cells were incubated for an additional 4 h. Then we removed the supernatant and used 150 μL dimethyl sulfoxide to dissolve the formed formazan crystals. At last, we used a Bio‐Rad‐550 (Bio‐Rad Laboratories, Redmond, WA, USA) microplate reader to detect the absorbance of each well at 490 nm (*A*_490 nm_). The experiment was repeated three times.

### Cell migration and invasion assays

#### Wound healing assay

HuH‐7 and Hep G2 cells (5 × 10^6^ cells per well) were inoculated into a six‐well plate. After cells were grown to 80% in confluence, a scratch was made on the center of cells using the tip of a 200‐μL pipette. Thereafter, cells were temporarily washed twice by medium to remove the floating cells and then cultured for an additional 24 h in a fresh medium. The cell migration distance at 0 and 24 h was measured under a microscope.

#### Transwell assay

A 24‐well Transwell chamber (8‐μm pores) from BD was used to identify cell invasion. We applied Matrigel to the upper chamber and placed about 2 × 10^4^ cells in it, and then added DMEM containing 10% FBS to the lower chamber. We cultured the cells in a 37 °C incubator for 48 h, then used a cotton swab to remove noninvading cells in the upper chamber and stained the cells that invaded to the lower chamber with 0.5% crystal violet. Finally, we observed the cells under a microscope and took pictures.

### Statistical analysis

We used spss 22.0 software (SPSS, Chicago, IL, USA) to process all data. All measurement data were expressed as mean ± standard deviation (SD). Student's *t*‐test and one‐way ANOVA were used to analyze the differences between two groups and the comparisons among more than two groups, respectively: *^,#^ *P* < 0.05, **^,##^ *P* < 0.01.

## Results

### *PZP* gene is hypermethylated in HCC tissue

**Fig. 1:**
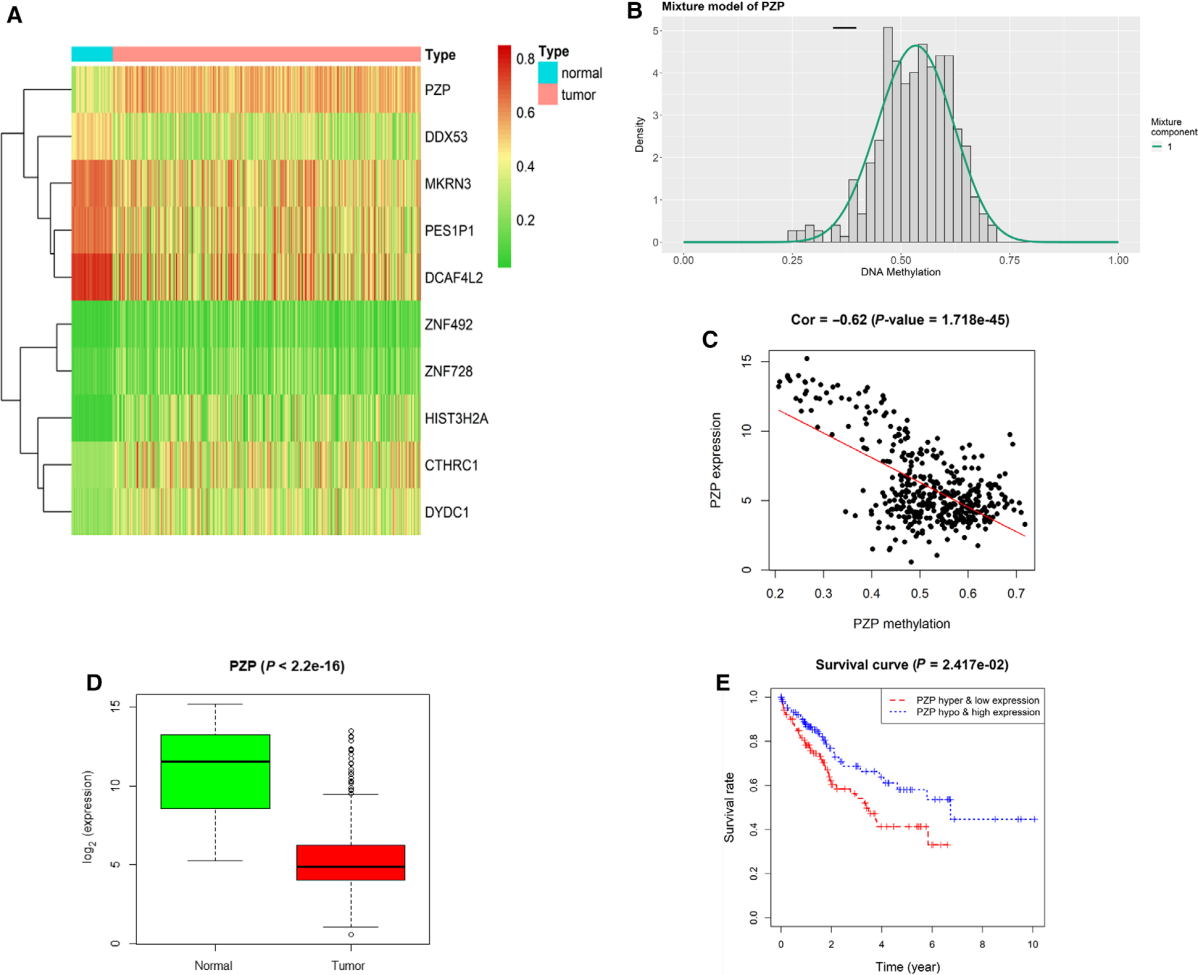
Analysis of methylation‐driven genes and prognosis of HCC. (A) Heatmap for HCC‐related methylation‐driven genes (the color from green to red stands for the tendency of methylation level from low to high). (B) Mixture model of *PZP* gene DNA methylation. The distribution map shows the methylation of *PZP* gene. The histogram and black horizontal bars, respectively, represent the distribution of methylation in tumor samples and in normal samples. (C) Correlation analysis for *PZP* methylation level and expression level. (D) Relative expression of *PZP* gene in TCGA‐LIHC database. (E) Survival analysis based on the methylation and expression levels of *PZP* gene.

To obtain the key gene that affects the occurrence and development of HCC in a DNA methylation manner, we downloaded methylation and mRNA expression data of HCC from the TCGA‐LIHC database. Ten candidate methylation‐driven genes were screened using the ‘MethylMix’ package after standardized treatment (Fig. 1A), among which *PZP* was markedly highly methylated (Fig. 1B) and its expression in HCC tissue was abnormally decreased (Fig. 1D). Further, the correlation analysis results suggested that *PZP* methylation level was markedly negatively correlated with its expression level (Fig. 1C). Survival analysis was performed based on the methylation and expression levels of *PZP*, unveiling that the survival time of patients with *PZP* hypermethylation and low expression was markedly lower than that of patients with low methylation and high expression (Fig. 1E). Accordingly, it can be concluded that *PZP* gene is hypermethylated in HCC tissue.

### CpG methylation of *PZP* gene in HCC and normal cells

**Fig. 2:**
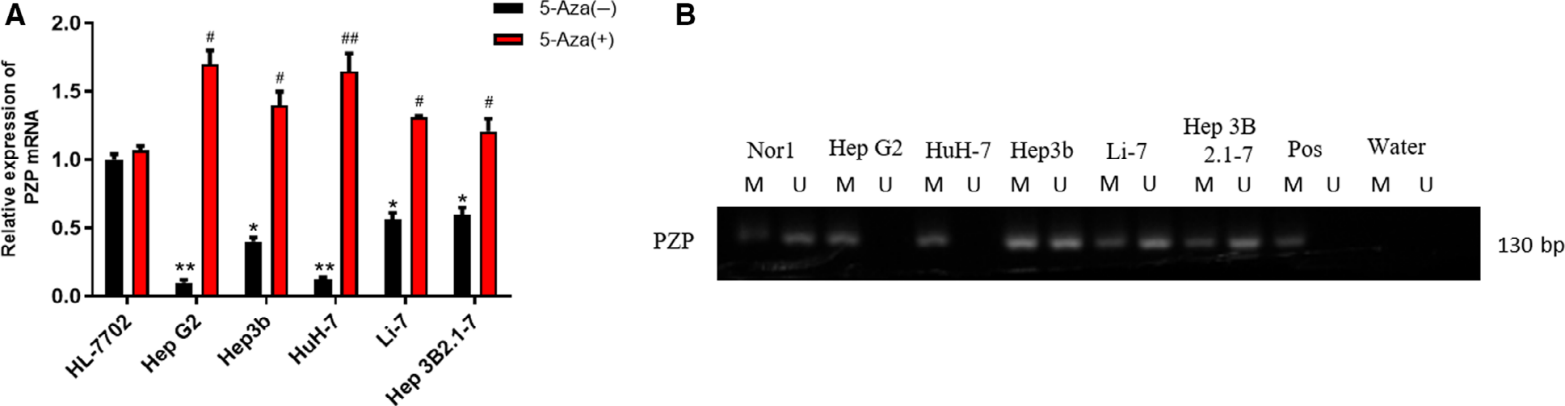
*PZP* expression and methylation in HCC cells. (A) Quantitative real‐time PCR was performed to detect mRNA expression of *PZP* in human liver cell line and five human HCC cell lines (asterisks [*] represent the comparison of *PZP* expression in normal liver cells and HCC cells; number signs [#] represent comparison of *PZP* expression between the HCC cells and that with or without 5‐Aza). (B) MSP result of *PZP* in HCC cell lines. Error bars indicate SD. All experiments were repeated three times. *P < 0.05 and **P < 0.01 represent a comparison with the 5‐Aza (–) treatment group of the HL‐7702 cell line; ^#^ *P* < 0.05 and ^##^ *P* < 0.01 represent a comparison with the 5‐Aza (–) treatment group of the corresponding cell line. The significance analysis of the results in Fig. 3A used a *t*‐test for the comparison of the results of each cell line with the 5‐Aza (–) treatment group and used ANOVA for the comparison with the 5‐Aza (–) treatment group of the HL‐7702 cell line. M, methylated allele; NorL, lymphocyte DNA used as the reference of nonmethylation; Pos, *in vitro* methylated DNA used as the reference of methylation; U, unmethylated allele.

Bioinformatics analysis indicated that *PZP* down‐regulation in HCC might be caused by its hypermethylation. Hence we further explored *PZP* methylation by conducting *in vitro* experiments. Firstly, quantitative real‐time PCR was performed to detect *PZP* mRNA expression in a human normal liver cell line and five HCC cell lines, and we discovered that *PZP* expression in HCC cells was lower than that in human normal cells, among which *PZP* was most markedly down‐regulated in Hep G2 and HuH‐7 cells (Fig. 2A). Subsequently, genomic DNAs of HCC cell lines were processed by sodium bisulfite, and *PZP* methylation was evaluated by MSP. *PZP* gene was amplified by methylation‐specific primers in Hep G2 and HuH‐7 cells but could not be done by nonmethylation‐specific ones, which uncovered that *PZP* was completely methylated in Hep G2 and HuH‐7 cells. In addition, *PZP* gene was amplified by both methylation‐specific primers and nonmethylation‐specific ones in Hep3b, Li‐7 and Hep 3B2.1‐7 cells, which demonstrated that *PZP* was partially methylated in these cells (Fig. 2B). After cell lines were treated with demethylating agents (5‐aza‐dC), quantitative real‐time PCR was performed and revealed that *PZP* expression was significantly increased in Hep G2, Hep3b, HuH‐7, Li‐7 and Hep 3B2.1‐7 cell lines by comparison with that in cells without 5‐aza‐dC (Fig. 2A), suggesting that *PZP* expression was regulated by its methylation. The experimental result was basically consistent with that of HCC methylation data in the TCGA‐LIHC dataset, which unveiled that *PZP* promoter hypermethylation inhibited *PZP* expression.

### *PZP* inhibits HCC cell proliferation, invasion and migration upon overexpression

**Fig. 3:**
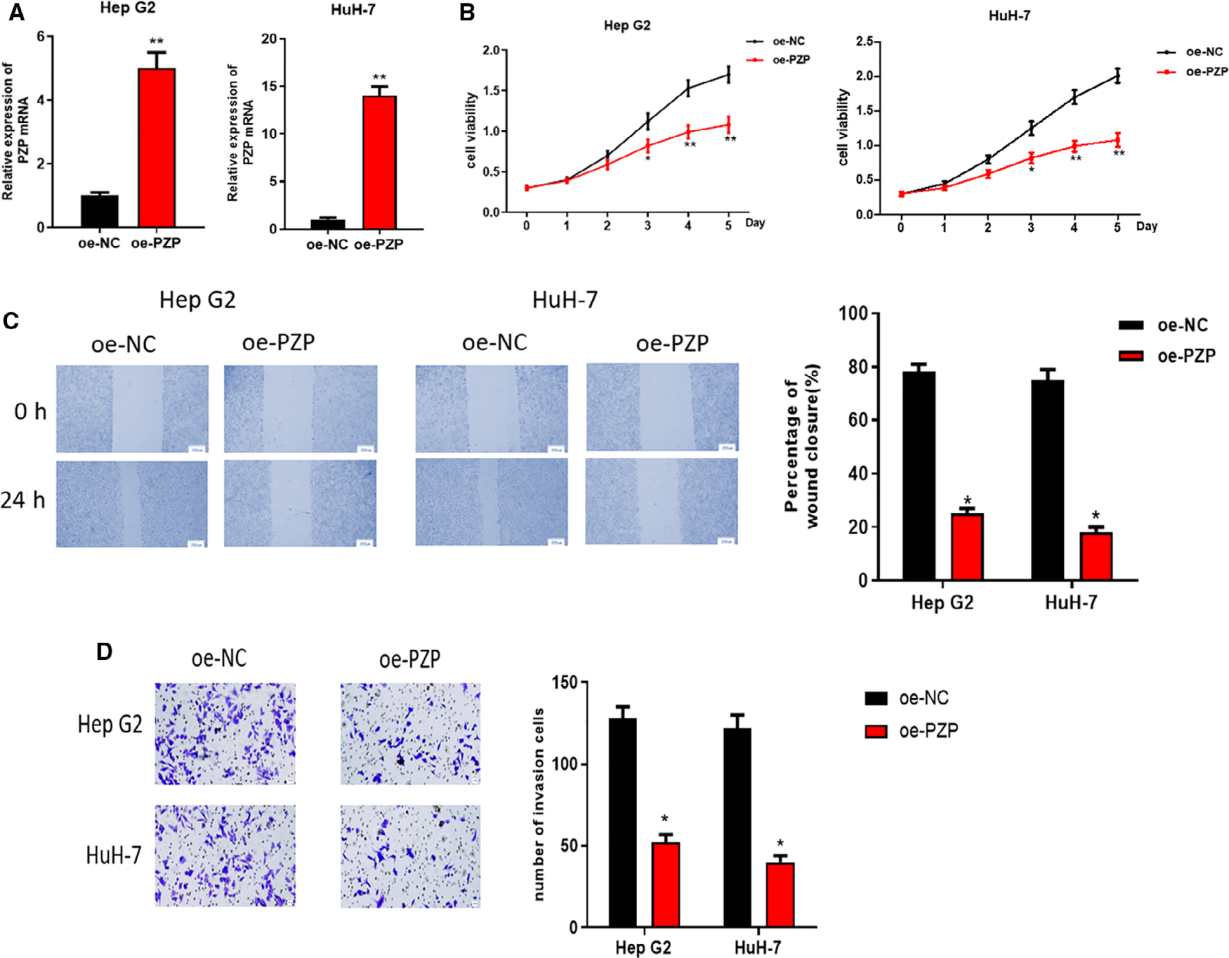
The effect of *PZP* on HepG2 and HuH‐7 cell growth. HepG2 and HuH‐7 cells were transfected with *PZP* overexpression vectors. (A) Quantitative real‐time PCR was conducted to detect *PZP* overexpression efficiency. (B) MTT assay was used to determine the viability of HepG2 and HuH‐7 cells transfected with pCDH‐*PZP* overexpression vector. (C) HepG2 and HuH‐7 cell migration upon *PZP* overexpression detected by wound healing assay. The statistical graph (right) shows the corresponding cell migration rate, scale: 200 µm. (D) Cell invasion graph of HepG2 and HuH‐7 cells breaking through Matrigel in Transwell assay. Scale bars: 20 µm. The statistical graph (right) shows the relative cell invasion number. Error bars indicate SD. All experiments were repeated three times. *P < 0.05 represents a comparison with the oe‐NC group, and **P < 0.01 represents a comparison with the oe‐NC group, Student's *t*‐test method. oe, overexpression.

In the earlier experiments, we found that *PZP* in HCC tissue was hypermethylated and down‐regulated. Thereafter, single‐gene Gene Ontology enrichment analysis for the *PZP* gene found that *PZP* was mainly involved in the biological processes, such as protein complement cascade activation and coagulation regulation. The results are shown in Table S2. Because ‘complement and coagulation cascade’ may be associated with the metastasis of clear cell renal cell carcinoma (CCRCC) [15], and the complement system plays a key role in tumor progression and can enhance angiogenesis and promote tumor growth, as well as metastasis [16], we investigated the effect of *PZP* on HCC cell proliferation, invasion and migration *in vitro*. We performed quantitative real‐time PCR on the HepG2 and HuH‐7 cells with pCDH empty vector and pCDH‐*PZP* overexpression vector to detect *PZP* mRNA expression and found that *PZP* expression in HCC cells with overexpressed *PZP* was obviously higher than that of the control (Fig. 3A). MTT assay was conducted and indicated that compared with the control group, the increase of *A*_490 nm_ of HepG2 and HuH‐7 cells upon *PZP* overexpression was significantly reduced (Fig. 3B), which suggested that cell proliferation ability was significantly decreased upon *PZP* overexpression. Wound healing and Transwell assays were used to further explore the effect of *PZP* on HCC cell invasion and migration upon overexpression. Compared with the control group, the wound healing rate and the number of HepG2 and HuH‐7 cells breaking through the membrane upon *PZP* overexpression were remarkably reduced, uncovering that the migratory and invasive abilities of the two cells were markedly weakened upon *PZP* overexpression (Fig. 3C,D). Collectively, the proliferative, migratory and invasive abilities of HepG2 and HuH‐7 cells could be markedly suppressed by overexpressing *PZP*.

## Discussion

At present, aberrant CGI methylation in the gene promoter region has become the hotspot of tumor research [7]. It contributes to activation of proto‐oncogenes or silencing of tumor suppressor genes, resulting in differential expression of downstream key genes, which leads to promotion of aberrant cell proliferation [6]. Due to its reversibility, DNA methylation serving as a therapeutic target of tumors will be promising, and the research of DNA methylation in HCC also will draw extensive attention.

Currently, it has been found that many DNAs in HCC are abnormally methylated and affect the development of cancer. Research has indicated that Mybbp1a is able to combine with DNMT1 to form a complex, can induce aberrant CGI hypermethylation of Igfbp5 to inhibit the secretion of Igfbp5, and can further activate IGF1/AKT signaling pathway and facilitate the occurrence and development of HCC [17]. Besides, HOTAIR represses miR‐122 expression, activates Cyclin G1 protein expression and promotes HCC tumor formation by epigenetic inheritance of DNA methylation [18]. Besides, osteopontin changes DNA methylation by up‐regulating DNMT1, which makes CD133^+^/CD44^+^ tumor stem cells in HCC sensitive to 5‐aza‐dC [19].

In this study, 10 candidate methylation‐driven genes were screened through the TCGA‐LIHC database, among which *PZP* was markedly hypermethylated in HCC tissue and its methylation level was markedly negatively correlated with its expression level. Survival analysis was performed based on the methylation and expression levels of *PZP*, unveiling that the survival time of patients with *PZP* hypermethylation and low expression was markedly shorter than that of patients with low methylation and high expression. Consequently, we speculated that methylation of the *PZP* gene in the promoter region inhibits the binding of certain transcription factors, hence leading to transcriptional repression. Next, cells were further processed by a demethylating agent 5‐azacytidine (5‐Aza), which unveiled that demethylation inhibitor could demethylate *PZP* to further activate gene expression. Thereafter, we discovered that overexpressing *PZP* could suppress HCC cell proliferation, invasion and migration. Taken together, the results of our experiments verified that *PZP* acts as a tumor suppressor gene, and its hypermethylation promotes HCC cell proliferation, migration and invasion. The finding helps people better understand the role of *PZP* methylation in HCC and provide a basis for exploring novel therapeutic targets for HCC.

## Conflict of interest

The authors declare no conflict of interest.

## Author contributions

All authors contributed to data analysis and drafting and revising the article, gave final approval of the version to be published and agreed to be accountable for all aspects of the work.

## Supporting information


**Table S1.** Primer sequences.

**Table S2**. Results of GO enrichment analysis.

## Data Availability

The data and materials in this study are available from the corresponding author upon reasonable request.
