# Supplementary material for: Hypermethylation of the PZP gene is associated with hepatocellular carcinoma cell proliferation, invasion and migration
Source: FEBS Open Bio. 2021 Feb 21;11(3):826–32. doi: 10.1002/2211-5463.13093 (PMC7931217; doi:10.1002/2211-5463.13093)
Supplement: Supplementary file 1 — Table S1. Primer sequences. [file FEB4-11-826-s001.docx]

| **MSP primer** | **sense sequence** **(5’-3’)** | **anti-sense sequence (5’-3’)** |
| --- | --- | --- |
| *PZP (M)* | GGAAGTTTTTAGAGTTTTAAATCGG | AAATTAAACTCCTTATTTCTACTTTCTACG |
| *PZP (U)* | GGAAGTTTTTAGAGTTTTAAATTGG | AATTAAACTCCTTATTTCTACTTTCTACAC |
| **qRT-PCR** | **sense sequence (5’-3’)** | **anti-sense sequence (5’-3’)** |
| *PZP* | CACACTGAGGCCCCTAAGAA | TCTCAGTGTTGCCCCAAACA |

**Supplementary Table 1 Primer sequences**
